# Affective Personality Traits in Olfactory Dysfunction: the Role of Dysthymia and Arousal

**DOI:** 10.1007/s12078-017-9242-6

**Published:** 2017-12-14

**Authors:** Anne Schienle, Axel Wolf, Peter Valentin Tomazic, Rottraut Ille

**Affiliations:** 1 Department of Psychology, University of Graz, Universitätsplatz 2, 8010 Graz, Austria; 2 BioTechMed-Graz, Graz, Austria; 3 Department of Otorhinolaryngology, Head and Neck Surgery, Medical University of Graz, Auenbruggerplatz 26, A-8036 Graz, Austria

**Keywords:** Anosmia, Hyposmia, Trait depression, Trait anxiety

## Abstract

**Introduction:**

Olfactory dysfunction can have a negative impact on emotional well-being. The aim of the present study was to examine associations between olfactory deficits and two affective personality characteristics (trait anxiety/trait depression).

**Methods:**

A questionnaire study was conducted with a total of 116 participants (33 classified as anosmic, 40 as hyposmic, and 39 as normosmic). All participants gave self-reports on two facets of trait depression (dysthymia, euthymia) and trait anxiety (arousal, worrying). Due to the fact that in all three groups, trait depression and anxiety were substantially correlated, analyses of covariance were conducted.

**Results:**

After controlling for trait depression, anosmic and hyposmic patients showed lower trait arousal compared to normosmic controls (partial *η* ^2^ = .05). After controlling for trait anxiety, patients scored higher on dysthymia (partial *η* ^2^ = .06).

**Conclusions:**

This study underlines the importance of statistically isolating specific associations between each of these affective personality characteristics and olfactory dysfunction.

**Implications:**

The present findings suggest that olfactory dysfunction can have opposite effects on facets of trait depression and trait anxiety.

## Introduction

Numerous studies have shown that olfactory dysfunction can have a negative impact on quality of life as reflected by difficulties in different areas, such as reduced appreciation of food and drink, problems concerning social/sexual relationships, concerns about personal hygiene, and difficulties in detecting environmental hazards (e.g., smoke, spoiled food). It has been suggested that these restrictions and constraints are likely to predispose a person with a smell disorder to experiencing a depressed mood (e.g., Neuland et al. 2011; Croy et al. 2012, 2014; Boesveldt et al. 2017). For example, Croy et al. (2012) investigated patients who were born without a sense of smell (congenital anosmia). These patients obtained higher mean scores (M ± SD) on the Beck Depression Inventory (BDI; Hautzinger et al. 1994) than normosmic controls (10.47 ± 9.38 vs. 4.63 ± 6.61). Lemogne et al. (2015) compared three groups (congenital anosmia, acquired anosmia, normosmia) and found slightly higher BDI scores in patients with both types of anosmia, relative to normosmic individuals (all groups were characterized by average BDI scores ≤ 10). A review and data combination of three studies (Kohli et al. 2016) on primary olfactory dysfunction and depression obtained similar findings. Mean BDI scores differed significantly between participants classified as normosmic, hyposmic, or anosmic (5.21 ± 4.73 vs. 10.93 ± 9.25 vs. 14.15 ± 5.39). It should be noted that BDI scores below 11 indicate no depression; a score of 18 or above points to the clinical relevance of depressive symptoms (tentative depression diagnosis; Hautzinger et al. 1994). Thus, anosmic/ hyposmic patients experienced affective changes (elevated sad mood) in their daily lives, however no depression in the sense of a mental disorder.

Investigations that focused on other affective personality characteristics in olfactory dysfunction, such as trait anxiety or trait disgust, are rare. In a questionnaire study by Ille et al. (2016), anosmic and hyposmic men answered a self-report measure on the personality trait of disgust proneness (tendency of a person to experience disgust in different areas of daily life; Schienle et al. 2002). The patients reported slightly lowered disgust to “spoilage/decay” (e.g., contact with spoiled food) and increased disgust to “poor hygiene” (e.g., seeing something dirty) relative to normosmic men.

Further, patients with congenital anosmia reported enhanced social anxiety in a study by Croy et al. (2012). However, Lemogne et al. (2015) found no statistically significant differences in trait anxiety between patients with congenital anosmia and healthy controls. Lehrner et al. (2015) reported elevated neuroticism scores for a group of dysosmic patients (relative to normosmic controls), pointing to the role of emotional instability in olfactory dysfunction. In summary, the results for trait anxiety found are conflicting. This might be partly due to the fact that it is not a homogenous construct. Laux et al. (2013) developed a personality questionnaire (trait section of the State-Trait Anxiety Depression Inventory) that assesses individuals’ tendency to experience anxiety (and depression) in their daily lives. The underlying model suggests that trait anxiety consists of two components: a cognitive component (Worrying) and an affective-somatic component (Arousal). Moreover, trait depression can be differentiated into Dysthymia (depressed mood) and Euthymia (positive mood), which is reduced in depression. The four components can be considered part of the broader personality construct neuroticism (Laux et al. 2013). Therefore, it is understandable that these components are not completely independent from each other.

The aim of the present study was to investigate the role of trait components of anxiety and depression in olfactory dysfunction (anosmia, hyposmia). It was hypothesized that anosmic/hyposmic patients would report elevated trait depression (dispositional sad mood) and elevated trait anxiety (disposition to experience situations as threatening) relative to normosmic individuals. Due to the substantial correlation between trait anxiety and trait depression, analyses of covariance were conducted in order to examine the specific association between the studied personality facets (worrying, arousal/ dysthymia, euthymia) and olfactory performance.

## Materials and Method

### Participants

Patients with anosmia (AN; *n* = 33), hyposmia (HYP; *n* = 40), and normosmic participants (NORM; *n* = 39) were included in the study. Mean age was 37.58 years (SD = 10.78). The three groups did not differ in age and gender distribution (*p*s ≥ .08). The majority of the sample was male (61.6%). Years of education were comparable in the patient groups and control group (*p* > .20; *M* = 11.61 years, SD = 3.72).

Physicians (specialists) at a Department of Otorhinolaryngology at a University Hospital examined all patients. The diagnoses anosmia and hyposmia were based on the results of a clinically approved test of olfactory function (extended Sniffin’Sticks test by Hummel et al. 2007). In addition, each patient was physically examined and the medical history was taken in order to identify the underlying causes of olfactory dysfunction. The causes were categorized as follows: sinunasal disease (40%; nasal polyps, sinusitis), non-sinunasal disease (31%; cranio-cerebral trauma, viral infections, such as influenza, olfactory meningioma), and idiopathy (29%, no medical cause identifiable). Duration of symptoms was on average 72 months (range 20–440).

The control subjects had been recruited via advertisements at the psychology department of the University or they were screened for olfactory dysfunction at the University Hospital, and were diagnosed as normosmic.

Exclusion criteria for both groups were neurodegenerative disorders (e.g., Morbus Parkinson, dementia), alcohol/ drug abuse, smoking, and pregnancy. A short standardized clinical interview (Margraf 1994, duration: approximately 15 min) was conducted in order to screen for mental disorders. None of the participants reported clinically relevant symptoms of depression or anxiety disorders (except for specific phobia).

The study was conducted in accordance with the Declaration of Helsinki and was approved by the ethics committee of the University. Written informed consent was obtained from each individual.

### Olfactory Measurement

Each participant was individually tested regarding olfactory threshold, discrimination, and identification capability with the extended Sniffin’Sticks test battery (Burghart ltd. Instruments, Wedel, Germany; Hummel et al. 2007). The odorants were presented to the blind-folded participants with pen-like odor-dispensing devices. The olfactory detection threshold was assessed with 푛-butanol, which was presented in 16 dilutions in a staircase, three-alternative, forced-choice procedure. Odor discrimination ability was obtained by presenting 16 triplets of odorant pens (two pens contain the same odorant; the third pen contains a different odorant). The participants’ task was to detect the different odor. Odor identification was assessed by means of 16 common odors (e.g., coffee). Subjects identified the odors by selecting the best label from a list of four descriptors. Possible scores for the detection threshold range between 1 and 16 (with higher scores indexing lower thresholds), and for the other two subtests between 0 and 16. The scores for all three subtests were summed to obtain the Threshold-Detection-Identification (TDI) score.

The groups differed on all measures of olfactory performance: threshold (*F*(2109) = 9.84, *p* < .001), discrimination (*F*(2109) = 161.09, *p* < .001), identification (*F*(2109) = 192.56, *p* < .001), and the TDI (*F*(2109) = 463.88, *p* < .001), with the AN group always scoring the lowest, followed by HYP group and the NORM group. All post-hoc comparisons were statistically significant (all *p*s < .001).

### State-Trait Anxiety Depression Inventory

All participants answered a personality questionnaire: the trait section of the State-Trait Anxiety Depression Inventory (STADI) by Laux et al. (2013). This section consists of four scales, each with five items. Trait Anxiety is comprised of “Trait Arousal” (e.g., I’m getting nervous quickly) and “Trait Worrying,” (e.g., I worry that something might happen); Trait Depression is determined based on the two subscales Trait Dysthymia (e.g., I am sad) and Trait Euthymia (e.g., I enyoy life). The items are rated on 4-point scales (1 = not all all, 4 = very much; scores for Euthmymia are inverted). Higher sum scores suggest higher levels of trait anxiety/ trait depression. The constructs can be viewed as relatively enduring dispositions of a person to feel nervousness and worry (trait anxiety) or to experience sad mood and a lack of positive mood (trait depression). The Cronbach’s alpha for the four subscales ranged between .79 and .88 in the present sample.

## Results

The descriptive statistics are presented in Table 1, separately for the three groups. When comparing the groups’ STADI scores to the construction sample means, there were significant differences for Euthymia. The NORM group (*t*(2243) = 4.62, *p* < .001), the HYP group (*t*(2244) = 4.53, *p* < .001), and the AN group (*t*(2237) = 2.81, *p* < .01) scored higher than the construction sample. For none of the other subscales, differences with the construction sample were found (*p*s > .10).

**Table 1:** Means and standard deviations for the trait scores of the State-Trait Anxiety Depression Inventory (STADI) and olfactory performance separately for three groups

	Normosmia (*n* = 39)	Hyposmia (*n* = 40)	Anosmia (*n* = 33)
	*M* (SD)	*M* (SD)	*M* (SD)
STADI			
Dysthymia	7.08 (2.29)	7.60 (2.41)	7.81 (2.67)
Euthymia	15.95 (2.71)	15.88 (2.76)	15.18 (3.00)
Arousal	10.15 (3.00)	9.10 (2.58)	9.51 (2.71)
Worrying	9.49 (4.03)	8.85 (2.89)	9.42 (2.56)
Sniffin’Sticks test			
Threshold	6.65 (1.63)	4.48 (2.22)	1.20 (0.59)
Discrimination	13.95 (1.38)	10.48 (1.87)	5.79 (2.47)
Identification	13.23 (1.58)	11.08 (2.45)	4.55 (1.54)
Total score	33.83 (2.02)	26.03 (3.99)	11.50 (3.00)

In all groups, trait anxiety and trait depression were significantly correlated (Pearson’s *r*) with each other (AN .702; HYP .559; NORM .475; all *p*s < .002).

### Group Differences in Euthymia and Dysthymia

Due to the substantial correlation between overall trait anxiety and trait depression scores, we conducted ANCOVAs, controlling for trait anxiety, in order to examine the unique relationship between trait depression and olfactory performance. The results showed that, when controlling for differences in trait anxiety, the three groups differed in Dysthymia (*F*(2108) = 3.59, *p* = .03, partial eta squared (ηp^2)^ = .06). Examination of the estimated marginal means (i.e., Dysthymia means adjusted for anxiety) showed that the adjusted Dysthymia means for the NORM group (*M* = 6.84, SE = .30) were lower compared to the HYP group (*M* = 7.86, SE = .30, *p* = .02) and the AN group (*M* = 7.79, SE = .32, *p* = .03). The HYP and AN group did not differ from each other (*p* = .86). After adjusting for trait anxiety, the groups did not differ in Euthymia (*F*(2108) = 1.01, *p* = .37). The effect of gender was not statistically significant (all *p*s > .35) and was therefore not included in the model.

### Group Differences in Worrying and Arousal

The ANCOVA which controlled for trait depression showed that the group differences were significant for Arousal (*F*(2108) = 2.95, *p* = .04, ηp^2^ = .05). Pairwise comparisons of the adjusted means showed that the NORM group had higher scores on the Arousal subscale (*M* = 10.34, SE = .39) compared to the HYP group (*M* = 9.12, SE = .39, *p* = .03) and marginally higher compared to the AN group (*M* = 9.27, SE = .43, *p* = .07). The AN group and HYP group did not differ statistically significant (*p* = .79). Differences in Worrying were not statistically significant after controlling for trait depression (*F*(2108) = .96, *p* = .39). The effect of gender was not statistically significant (all *p*s > .21) and was therefore not included in the model.

## Discussion

This study assessed components of trait anxiety (Worrying, Arousal) and trait depression (Euthymia, Dysthymia) in individuals with a loss or decrease in olfactory function. The main finding was that facets of trait depression (Dysthymia) and trait anxiety (Arousal) showed an association with olfactory dysfunction when controlling for either trait depression/ anxiety. It is of note that depression and anxiety were highly correlated with each other, which has not been taken into account in past research on olfactory dysfunction. The typical approach consists of comparing patient groups and healthy controls on one affective measure (e.g., depression). The ANCOVA approach revealed differences in Dysthymia between the groups only after controlling for trait anxiety; the expected pattern with more negative mood in patients did indeed then appear (e.g., Croy et al. 2012; Kohli et al. 2016).

A very interesting effect emerged with regard to Arousal. After controlling for trait depression, it became obvious that the patients reported lower Arousal (nervousness, somatic anxiety symptoms) compared to the healthy controls. Previous research has found no statistically significant differences in trait anxiety between patients with anosmia (acquired, congenital) and healthy controls, but it is important to note that that study did not apply an ANCOVA approach (Lemogne et al. 2015). However, reduced anxiety due to olfactory deficits has been observed in animal models on anosmia (e.g., Ahn et al. 2016). After the destruction of the olfactory epithelium in mice, the “open field test” (exploration of a novel environment) was conducted to assess anxiety-related behavior: it was found that the procedure did have an anxiolytic effect. The mice showed more exploration (and less avoidance), because there were no longer any olfactory alarm signals in the environment. Such warning cues are processed automatically and lead to autonomic nervous system activation (in order to initiate defensive behavior). Moreover, the administration of anxiolytics in healthy animals increased their exploration behavior (Ahn et al. 2016). Critically, it has to be mentioned that an effect seen in macrosmatic rodents might not directly relate to microsmatic humans. But it seems plausible that a prolonged deficit in perceiving olfactory threat signals might reduce certain aspects of trait anxiety (e.g., bodily arousal, nervousness).

It is important to emphasize that in the current study, neither hyposmic nor anosmic patients reported above-average trait depression and trait anxiety. Comparisons of the scores with the construction sample of the STADI (Laux et al. 2013) indicated no differences for Arousal, Worrying, and Dysthymia. For Euthymia, all groups studied were actually characterized by slightly elevated scores, reflecting positive mood. Thus, despite their sensory disability and associated difficulties in daily life (e.g., reduced enjoyment of food, concerns about personal hygiene) this did not enhance trait anxiety/depression to a clinically relevant level in the patients. The findings for depression are in line with previous reports, which have also identified only minor to mild depressive complaints in anosmic and hyposmic patients on average (e.g., Croy et al. 2012, Kohli et al. 2016). Pertaining to the present patient sample, it is possible that Euthymia acted as a buffer or protecting factor. Future studies on personality traits should also incorporate “positive” personality factors in order to identify their possible moderating role for coping with olfactory impairment.

As a shortcoming of the present investigation, it has to be noted that we did not study a representative group of patients with olfactory impairment, but instead individuals who agreed to partake in the research project after being diagnosed at the University hospital. Investigations with different approaches (e.g., surveys in self-help groups) have found significant complaints of depression and anxiety (e.g., Philpott and Boak 2014). However, for those types of studies not using detailed somatic/psychological diagnostics, it cannot be ruled out that the patients had a primary diagnosis of a mental disorder (e.g., primary depression), or that comorbid conditions had led to increased depression or anxiety.

## Conclusion

The present findings suggest that olfactory dysfunction can have opposite effects on specific components of trait depression and trait anxiety.
